# Decoupling of regional neural activity and inter-regional functional connectivity in Alzheimer’s disease: a simultaneous PET/MR study

**DOI:** 10.1007/s00259-022-05692-1

**Published:** 2022-02-24

**Authors:** Somayeh Maleki Balajoo, Farzaneh Rahmani, Reza Khosrowabadi, Chun Meng, Simon B. Eickhoff, Timo Grimmer, Mojtaba Zarei, Alexander Drzezga, Christian Sorg, Masoud Tahmasian

**Affiliations:** 1 Institute of Medical Science and Technology, Shahid Beheshti University, Tehran, Iran; 2 Institute of Systems Neuroscience, Heinrich Heine University Düsseldorf, Düsseldorf, Germany; 3 Institute of Neuroscience and Medicine, Brain & Behaviour (INM-7), Research Centre Jülich, Jülich, Germany; 4 Department of Radiology, Washington University School of Medicine, St. Louis, MO 63110 USA; 5 Institute for Cognitive and Brain Sciences, Shahid Beheshti University, Tehran, Iran; 6 The Clinical Hospital of Chengdu Brain Science Institute, MOE Key Laboratory for Neuroinformation, Center for Information in Medicine, School of Life Science and Technology, University of Electronic Science and Technology of China, Chengdu, 611731 China; 7 Department of Psychiatry and Psychotherapy, Klinikum Rechts Der Isar, Technical University of Munich, Munich, Germany; 8 Department of Neurology, Odense University Hospital, Odense, Denmark; 9 Department of Clinical Research, University of Southern Denmark, Odense, Denmark; 10 Department of Nuclear Medicine, Faculty of Medicine and University Hospital Cologne, University of Cologne, Cologne, Germany; 11 German Center for Neurodegenerative Diseases (DZNE), Bonn-Cologne, Germany; 12 Institute of Neuroscience and Medicine (INM-2), Molecular Organization of the Brain, Forschungszentrum Jülich, Jülich, Germany; 13 Department of Neuroradiology, Klinikum Rechts Der Isar, Technische Universität München, Munich, Germany; 14 Klinikum Rechts Der Isar, TUM-Neuroimaging Center (TUM-NIC), TechnischeUniversitätMünchen, Munich, Germany

**Keywords:** Alzheimer’s disease, Mild cognitive impairment, PET/MR, Neural activity, Functional connectivity, Graph analysis

## Abstract

**Purpose:**

Alzheimer’s disease (AD) and mild cognitive impairment (MCI) are characterized by both aberrant regional neural activity and disrupted inter-regional functional connectivity (FC). However, the effect of AD/MCI on the coupling between regional neural activity (measured by regional fluorodeoxyglucose imaging (rFDG)) and inter-regional FC (measured by resting-state functional magnetic resonance imaging (rs-fMRI)) is poorly understood.

**Methods:**

We scanned 19 patients with MCI, 33 patients with AD, and 26 healthy individuals by simultaneous FDG-PET/rs-fMRI and assessed rFDG and inter-regional FC metrics (i.e., clustering coefficient and degree centrality). Next, we examined the potential moderating effect of disease status (MCI or AD) on the link between rFDG and inter-regional FC metrics using hierarchical moderated multiple regression analysis. We also tested this effect by considering interaction between disease status and inter-regional FC metrics, as well as interaction between disease status and rFDG.

**Results:**

Our findings revealed that both rFDG and inter-regional FC metrics were disrupted in MCI and AD. Moreover, AD altered the relationship between rFDG and inter-regional FC metrics. In particular, we found that AD moderated the effect of inter-regional FC metrics of the caudate, parahippocampal gyrus, angular gyrus, supramarginal gyrus, frontal pole, inferior temporal gyrus, middle frontal, lateral occipital, supramarginal gyrus, precuneus, and thalamus on predicting their rFDG. On the other hand, AD moderated the effect of rFDG of the parietal operculum on predicting its inter-regional FC metric.

**Conclusion:**

Our findings demonstrated that AD decoupled the link between regional neural activity and functional segregation and global connectivity across particular brain regions.

**Supplementary Information:**

The online version contains supplementary material available at 10.1007/s00259-022-05692-1.

## Introduction

Alzheimer’s disease (AD) and mild cognitive impairment (MCI), a syndrome at risk for AD, are characterized by progressive cognitive dysfunctions due to neuronal loss and reduced overall neural activity, which can be indirectly quantified through fluorodeoxyglucose-positron emission tomography (FDG-PET) [1]. Regional glucose metabolism (rFDG) is associated with cortical atrophy and beta-amyloid/tau protein accumulation, which are hallmarks of AD pathology [2, 3]. As 80% of neural metabolic activity is dedicated to synaptic signaling [4], intrinsic functional connectivity (FC) measured by resting-state functional magnetic resonance imaging (rs-fMRI) might indicate a conjugate in-/decreased blood oxygenation level–dependent (BOLD) signaling between the brain regions. Topological features of these FC networks can be modeled through graph theory analyses, in which brain regions are considered “nodes,” and FC between them as “edges” of the graph [5]. Several studies demonstrated FC alterations and topological changes, mainly in the default mode network (DMN), along the trajectory of AD [6–9]. However, the effect of AD/MCI on the coupling between regional neural activity and inter-regional FC metrics is not well-addressed in literature. Simultaneous assessment of rFDG and FC has a great potential to identify how disease can alter their coupling, which might shed light on the neurobiological mechanisms of AD.

Empirical evidence and computational modeling [10–12] suggested that higher local metabolism (as a proxy for local activity) in a particular brain region increases its sensitivity to afferent input from other regions and thus determines the likelihood of inter-regional FC (as a proxy for synchronous fluctuations of BOLD signal between the brain regions). A recent study found synchrony and coupling between the neural dynamics of glucose metabolism and the BOLD hemodynamic response [13], suggesting a strong relationship between rFDG and FC. In healthy brains, up to 18% of inter-subject variabilities in whole-brain glucose metabolism can be explained by differences in FC [14]. Moreover, it has been shown that increased FC and higher functional clustering are associated with a non-linear increase in regional metabolism in healthy individuals [14]. This association, however, could be disturbed in AD, where rFDG is no longer linked with regional FC in the posterior associational cortices [15]. Here, we aimed to address whether and how AD/MCI (as a clinical diagnosis of disease status) affects the coupling between rFDG and inter-regional FC metrics characteristics using simultaneous acquisition of FDG-PET and rs-fMRI in healthy control (HC) subjects and patients with MCI and AD. Initial results of this study were already uploaded as a preprint in BioRxiv (https://doi.org/10.1101/642629).

## Methods

### Participants

Thirty-three patients with mild AD-dementia, 19 patients with MCI, and 26 HC subjects were recruited in this cross-sectional study. Patients were randomly selected from outpatient memory clinic of the Department of Psychiatry and Psychotherapy of Klinikum rechts der Isar, Technical University of Munich (TUM). Diagnosis of AD or MCI was determined using Clinical Dementia Rating (CDR) and neuropsychological testing batteries based on criteria established by Consortium to Establish a Registry for Alzheimer’s disease CERAD. HC subjects were also recruited through word-of-mouth advertising in Munich. This study was approved by the TUM ethics committee in line with the institute’s Human Research Committee guidelines and conformed to standards of the declaration of Helsinki. Written informed consent was obtained from all participants after providing detailed information about this study. Of note, as our AD/MCI patients did not have amyloid-/tau-PET imaging, “AD/MCI effect” solely refers to the presence or absence of clinical diagnosis here.

### Data acquisition and preprocessing

Imaging data acquisition included structural MRI (T1 weighted), rs-fMRI, and FDG-PET, which were simultaneously acquired on a Biograph hybrid PET/MR scanner (Siemens, Erlangen, Germany). Images were preprocessed and analyzed according to previously published and standardized protocols (Supplemental Methods, Sect. 1) [8, 16, 17]. After removing eight subjects due to excessive motion (> 2 mm translation or 2° rotation), 30 patients with mild AD-dementia, 16 patients with MCI, and 24 HC subjects were included for further analyses (Table 1).

**Table 1:** Demographic and clinical data of included participants

	HC	MCI	AD	*p*-value
Mean age, year *(SD)*	54.71 (9.99)	70.19 (6.65)	70.5 (8.50)	< 0.001
*N (female)*	24 (9)	16 (12)	30 (11)	0.03
Mean MMSE score *(SD)*	29.44 (1.10)	26.64 (2.20)	22.68 (4.40)	< 0.001
Mean CREAD NAB total *(SD)*	86 (8.23)	67.89 (11.54)	53.67 (11.94)	< 0.001

Abbreviations: *HC*, healthy control; *MCI*, mild cognitive impairment; *AD*, Alzheimer disease; *MMSE*, Mini-Mental State Examination; *CERAD*, Consortium to Establish a Registry for Alzheimer’s Disease neuropsychological assessment battery. Group comparisons were done based on analysis of variance, *p* < 0.05 as a threshold of significance, except for sex (Kruskal–Wallis test)

### FDG-PET data analysis

The average glucose uptake was computed for 112 cortical and subcortical regions obtained from Harvard–Oxford atlas [18] in MNI space from each subject-specific preprocessed FDG-PET data. Afterward, we normalized the mean glucose uptake value of each region by dividing it to the average glucose uptake of the pons of the same subject, as choosing the pons (as a reference region) is sensitive to detect hypometabolic changes associated with AD [19, 20].

### rs-fMRI data analysis

The average BOLD signal of 112 regions from Harvard–Oxford atlas was extracted, and the inter-regional FCs were modeled based on graph theory analysis, where the atlas regions considered as nodes and FC between them (using absolute values of Pearson’s correlation coefficients) as edges. Finally, a 112 × 112 symmetric undirected weighted matrix representing individual whole-brain inter-regional FC was computed for each subject. From a topological perspective, each subject has a different topology in terms of connection density between the brain regions. The connection density in a graph is defined as a ratio of number of existing edges to all possible edges [21]. Difference in connection density, in turn, influences most of the extracted topological metrics within a graph [22]. Thus, it is necessary to implement a matching strategy between FC graphs, prior to statistical analyses between the three groups [23]. Accordingly, we thresholded adjacency matrix of each subject for density range from 0.01 to 0.40 (with intervals of 0.01), as suggested earlier [24]. It means that the adjacency matrix of each subject was thresholded 40 times with different connection densities to ensure that the topology variability across all subjects was considered in the analysis. We then characterized the organization of brain regions in terms of clustering coefficient (CC; segregated role of node) [25] and degree centrality (DC; centralized role of node) [26] (Supplemental Methods, Sect. 2). Of note, CC and DC are two commonly used inter-regional FC topological metrics, representing local segregation and centrality of individual nodes, respectively [27]. Put differently, CC quantifies how well a certain node has formed locally segregated processing modules (i.e., clusters) around itself, while DC gives a measure of the centrality of a node in terms of interacting with other nodes within and outside the regional clusters and how well it is contributing to regional segregation and global integration of the network [5]. To assess the inter-regional FC metrics, we calculated those metrics using the Brain Connectivity Toolbox [5]. Finally, the integral values of CC and DC across the all thresholded adjacency matrices for each subject were used for between groups’ comparison, as recommended previously [28].

### Group comparison and statistical analyses

Between groups’ comparisons based on the analysis of covariance (ANCOVA) on rFDG and inter-regional FC metrics (i.e., CC/DC) were performed for all 112 regions, while age and sex of subjects were considered the covariates of no-interest. The results were corrected for multiple comparisons using N-region statistical comparison, as described previously [29]. The significance threshold was calculated using 1/(number of regions), *p*-value < 1/112 = 0.009. Post hoc analyses were then conducted for rFDG, CC, and DC utilizing permutation test with 100,000 permutations, and significance threshold for post hoc test was calculated using *p*-value = 1/(number of tests) to correct for potential false-positive errors.

### Cross-modality analysis

**Fig. 1:**
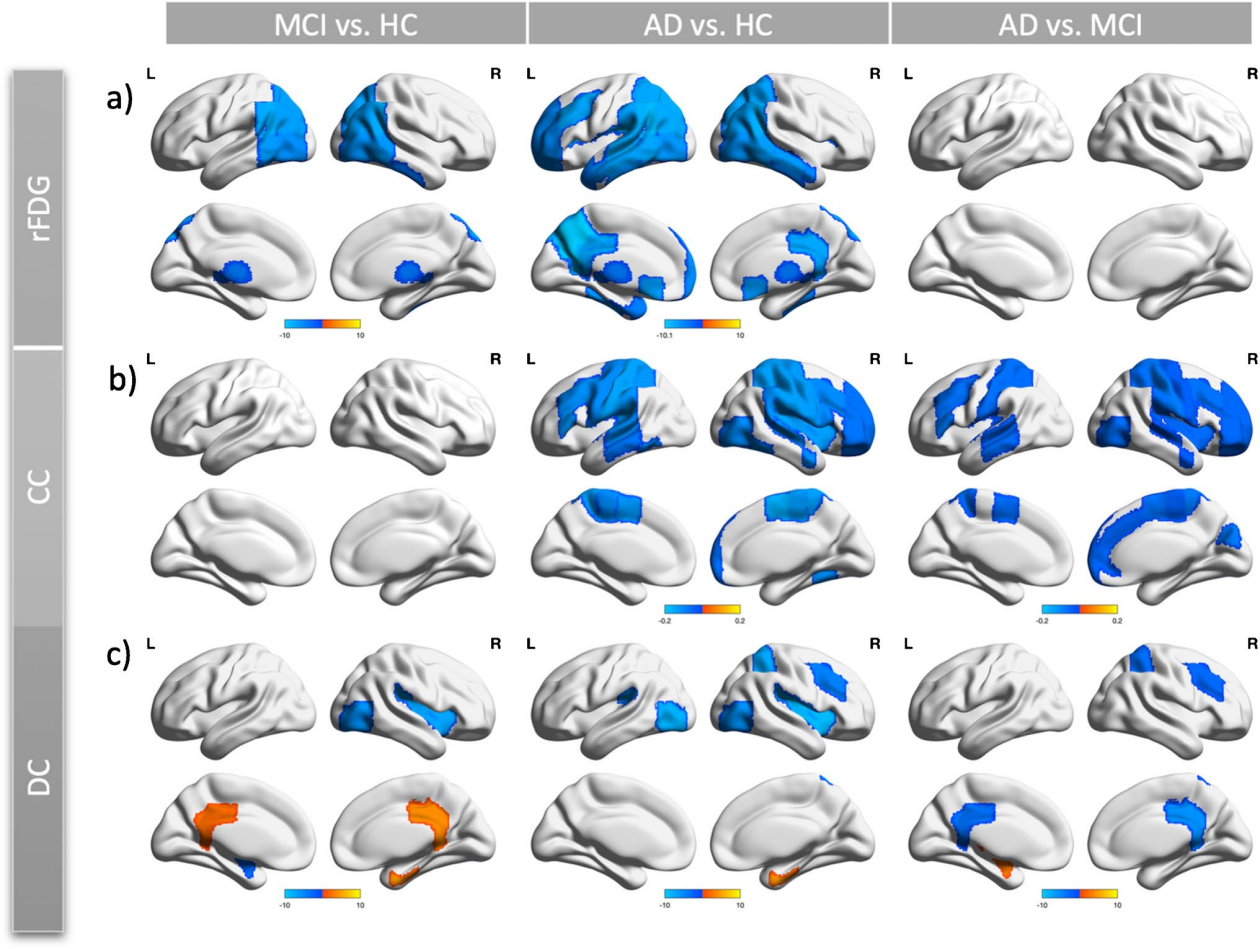
Analysis of covariance on whole-brain topological measures of inter-regional FC and regional glucose metabolism for each region in Harvard–Oxford Atlas in (a) regional glucose metabolism; (b) clustering coefficient; and (c) degree centrality. Age and sex were considered as covariates of no-interest. Post hoc test: permutation test (*p* < 0.05, 100,000 permutations). In each group comparison, the color of significant regions was coded as the mean difference values of rfDG/CC/DC between each pair of groups (i.e., in AD vs. HC: color code = CC_AD_ - CC_HC_). For more detailed information, see SI-Table 1-3. *FC*, functional connectivity; *AD*, Alzheimer disease; *HC*, healthy control; *MCI*, mild cognitive impairment; *CC*, clustering coefficient; *DC*, degree centrality; *rFDG*, regional glucose metabolism; *L*, left;* R*, right

To evaluate the moderating effect of disease (i.e., MCI/AD) on the link between rFDG the inter-regional FC metrics, we applied the hierarchical moderated multiple regression (HMMR) analysis (Supplemental Methods, Sect. 3; SI-Fig. 1) and focused on the regions in which rFDG or CC/DC were affected by diseases (Fig. 1). To assess our hypothesis about the effects of disease on the relationship between rFDG and inter-regional FC metrics, we tested this effect by considering interaction between disease status and inter-regional FC metrics and also interaction between disease status and rFDG. In the first model, both CC/DC and disease status and the interaction term as “CC/DC × disease status” were entered to a model to predict rFDG as a response variable. In the second model, rFDG and disease status and the interaction term as “rFDG × disease status” were entered to a model to predict CC/DC as a response variable. We also entered age and sex as the covariate of no-interest in the HMMR analyses. To evaluate whether the interaction term in both models is meaningful, we tested whether adding the interaction term increased the variance explained by the model in successive regression steps ($\Delta {R}^{2}$, according to the available guidelines [30, 31]). These results were also corrected for multiple comparisons using family wise error (FWE, *p*-value < 0.05).

## Results

### Regional glucose metabolism alterations along the trajectory of disease

Comparing rFDG between groups (controlling for age and sex), we found significantly reduced metabolism in the DMN areas including the bilateral posterior cingulate cortex, angular gyri, left parahippocampal gyrus, left middle frontal cortex, and left precuneus cortex in AD patients compared to HC subjects. There were also hypometabolism in AD patients compared to HC subjects in the subcortical regions (i.e., caudate, thalamus, nucleus accumbens), as well as in the temporal, lateral occipital, and frontal regions. We also found hypometabolism in the bilateral middle and inferior temporal gyri, bilateral lateral occipital gyri, bilateral angular gyri, and caudate in MCI patients compared to HC subjects (Fig. 1(a), SI-Table 1).

### Disrupted local FC network topology along the trajectory of disease

We observed widespread alterations in CC/DC metrics between the groups (Fig. 1(1), SI-Tables 2 and 3). In particular, there was a progressive decrease of CC along the trajectory of disease in several regions including the bilateral middle and inferior frontal, bilateral superior, middle and inferior temporal, bilateral supramarginal, bilateral supplementary motor, bilateral occipital, bilateral Heschel, bilateral post/precentral, right temporal occipital, bilateral operculum cortex, right insular, and left angular gyri (Fig. 1(1), SI-Table 2). CC was not significantly affected in MCI patients compared to HC subjects. Interestingly, in MCI patients, DC was increased in the bilateral posterior cingulate cortex (PCC) and right parahippocampal gyrus and decreased in the left amygdala and brainstem compared to both HC subjects and patients with AD. DC of right parahippocampal gyrus also increased in AD patients compared to HC subjects. Moreover, in both patients’ groups, DC was decreased in the right insular, bilateral lateral occipital, bilateral parietal operculum in bilateral precentral, lateral occipital, and bilateral Heschl’s gyri, and increased in the bilateral parahippocampal gyri (Fig. 1(1), SI-Table 3). The results indicated a pattern of progressive decrease in DC in the right hemisphere specifically in the superior parietal lobule, planum polare, middle frontal gyrus, parietal operculum, insular, and central opercular along the trajectory of disease (Fig. 1(1) and SI-Table 3).

### The moderator effect of diseases on the coupling between regional glucose metabolism and inter-regional FC metrics

#### Interaction between disease status and inter-regional FC metrics

**Fig. 2:**
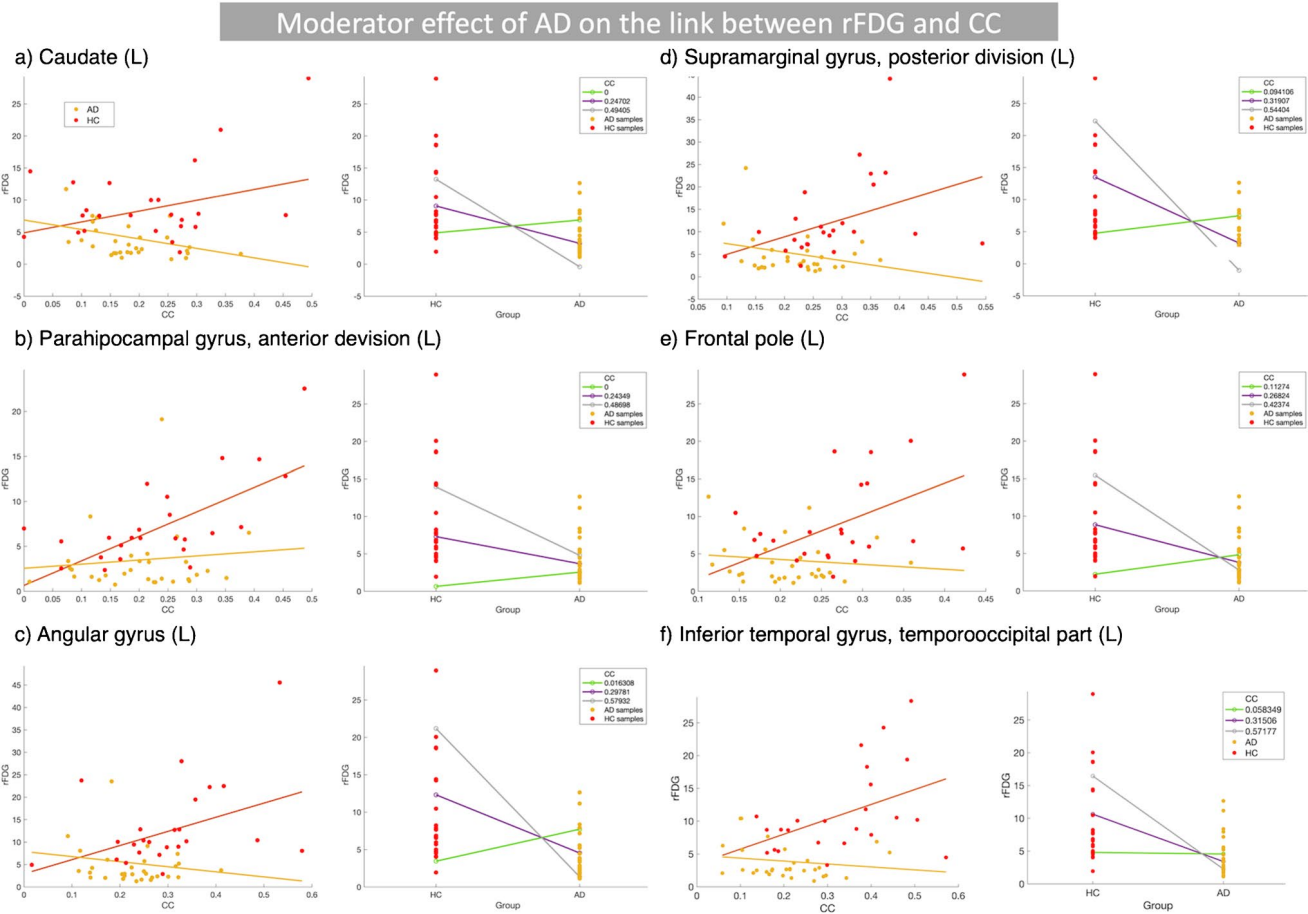
Moderator effect of AD on the link between regional glucose metabolism and inter-regional FC metrics by considering the interaction term between disease status and inter-regional FC metrics (CC) in HMMR model. (a-f) Brain regions where significant interactions between disease status and CC predicted rFDG changes (for more information, see Table 2). In each section, figures in the first column indicate that rFDG increases as a function of CC in HC subjects but decreases in AD patients. In the second column of each section, we showed changes in the rFDG in both HC and AD groups, when the CC is fixed at three low, medium, and high levels. The lines for HC and AD groups are crossing, indicating an interaction between CC and disease status. *AD*, Alzheimer’s disease; *HC*, healthy control; *HMMR*, hierarchical moderated multiple regression; *FC*, functional connectivity; *CC*, clustering coefficient; *rFDG*, regional glucose metabolism

In the first model, we considered the interaction between disease status and inter-regional FC metrics to test the potential effect of AD on the association between rFDG and CC/DC. There were significant two-way interactions between “disease status × CC” only in the left hemisphere including caudate, parahippocampal gyrus, angular gyrus, supramarginal gyrus, and frontal pole and inferior temporal gyrus that predicted changes in rFDG, when comparing the HC and AD groups. Thus, AD moderated the effect of CC of the abovementioned regions on predicting their regional glucose metabolism (Fig. 2(a-f); Table 2). Only left inferior temporal region showed both significant hypometabolism and decrease in CC in AD patients compared to HC subjects (SI-Table 4). rFDG showed a positive association with CC in HC subjects, but a negative association in AD patients (Fig. 2(a-f); Table 2). The significant interaction between CC and disease status indicated that the local properties of all these regions (rFDG and CC) depend on disease status. No significant interaction between disease status and CC predicted rFDG alteration, when comparing the AD vs. MCI or MCI vs. HC groups.

**Table 2:** Statistical results related to the first model considering the interaction between disease status and CC to predict rFDG changes, when comparing HC and AD groups

Model parameters and statistics		Caudate (L)	Parahipocampal, anterior (L)	Angular (L)	Supramarginal gyrus, posterior (L)	Frontal pole (L)	Inferior temporal, temporooccipital (L)
**HMMR model** ^**a,b**^	*R* ^2^	0.44	0.47	0.40	0.37	0.39	0.51
	Adjusted *R*^2^	0.38	0.42	0.34	0.31	0.33	0.46
	*F*(5,53)	7.50	8.60	6.50	5.66	6.16	10.01
	*P*-value_model (FWE-corrected)_	< 0.0001	< 0.0001	< 0.0001	< 0.0001	< 0.0001	< 0.0001
	$\Delta {R}^{2}$: *R*^2^ change	0.68	0.56	0.06	0.07	0.07	0.05
	*p*-value_interaction term_	0.02	0.03	0.04	0.02	0.03	0.03
**Standardized coefficients** ^**a**^	Disease status	0.19	0.20	0.30	0.51	0.74	0.11
	Age	− 0.21	− 0.14	− 0.16	− 0.10	− 0.10	− 0.073
	Sex	− 0.02	0.11	0.016	0.04	0.10	0.014
	CC	0.90	1.13	0.94	1.02	1.18	1.023
	Interaction (disease status $\times$ CC)	− 0.93	− 0.86	− 0.81	− 1.01	− 1.08	− 0.75

^a^Response variable: rFDG

^b^Predictors: CC, sex, age, disease status, interaction (disease status $\times$ CC)

Abbreviations: *AD*, Alzheimer’s disease; *HC*, healthy control; *HMMR*, hierarchical moderated multiple regression; *CC*, clustering coefficient; *rFDG*, regional glucose metabolism

**Table 3:** Statistical results related to the first model considering the interaction between disease status and DC to predict rFDG changes, when comparing HC and AD groups

Model parameters and statistics		Thalamus (L)	Thalamus (R)	Middle frontal gyrus (L)	Lateral occipital cortex, superior (L)	Supramarginal gyrus, posterior (L)	Precuneous cortex (L)
**HMMR model** ^**a,b**^	*R* ^2^	0.43	0.44	0.37	0.37	0.39	0.38
	Adjusted *R*^2^	0.37	0.38	0.31	0.30	0.33	0.31
	*F*(5,53)	7.31	7.39	5.69	5.61	6.19	5.78
	*P*-value_model (FWE-corrected)_	< 0.0001	< 0.0001	< 0.0001	< 0.0001	< 0.0001	< 0.0001
	$\Delta {R}^{2}$: *R*^2^ change	0.06	0.15	0.08	0.08	0.08	0.05
	*p*-value_interaction term_	0.03	0.001	0.02	0.02	0.01	0.05
**Standardized coefficients** ^**a**^	Disease status	0.06	0.26	0.23	0.23	0.24	0.22
	Age	− 0.17	− 0.12	− 0.17	− 0.04	− 0.07	− 0.09
	Sex	0.06	0.11	0.004	0.003	0.006	0.14
	DC	1.01	1.39	1.03	1.02	1.11	1.03
	Interaction (disease status $\times$ DC)	− 0.87	− 1.45	− 1.10	− 0.97	− 1.10	− 0.80

^a^Response variable: rFDG

^b^Predictors: DC, sex, age, disease status, interaction (disease status $\times$ DC)

Abbreviations: *AD*, Alzheimer’s disease; *HC*, healthy control; *HMMR*, hierarchical moderated multiple regression; *DC*, degree centrality; *rFDG*, regional glucose metabolism

**Fig. 3:**
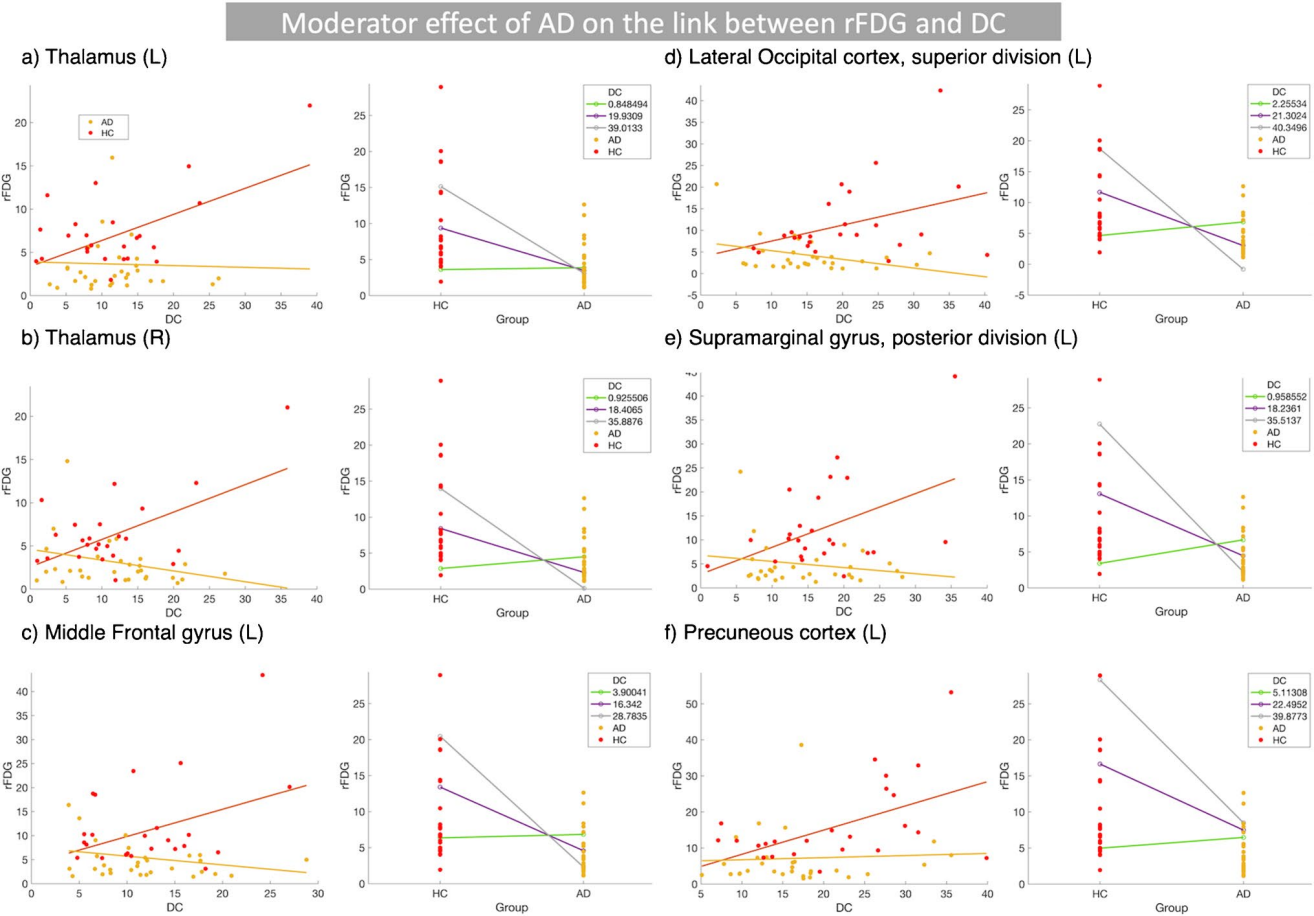
Moderator effect of AD on the link between regional glucose metabolism and inter-regional FC metrics by considering the interaction term between disease status and rFDG in HMMR model. Results showed that only in the left parietal operculum a significant interaction between disease status and rFDG predicted DC alteration, when comparing AD vs. HC groups (for more information, see Table 4). First column shows that DC decreases as a function of rFDG in HC subjects but increases in AD patients in the left parietal operculum. In second column, we presented that changes in the DC in both HC and AD groups, when the rFDG is fixed at three low, medium, and high levels. The lines for HC and AD groups are crossing, indicating an interaction between rFDG and disease status. *AD*, Alzheimer’s disease; *HC*, healthy control; *HMMR*, hierarchical moderated multiple regression; *FC*, functional connectivity; *DC*, degree centrality; *rFDG*, regional glucose metabolism

In addition, there were also significant two-way interactions between “disease status × DC” in the bilateral thalamus, left middle frontal, left lateral occipital, left supramarginal gyrus, and the left precuneus that predicted changes in rFDG, when comparing the HC and AD groups (Fig. 3(a-f); Table 3). Our results also demonstrated significant two-way interactions between “disease status × DC” in the bilateral thalamus that predicted changes in rFDG, when comparing the HC and MCI groups (SI-Fig. 2; SI-Table 5). Figures 3 (a-f) and SI-Fig. 2 indicate that rFDG showed a positive association with DC in HC subjects, but a negative association in AD/MCI patients. The significant interaction between DC and disease status indicated that the local properties of all these regions (rFDG and DC) depend on disease status. No significant interaction between disease status and DC could predict rFDG alteration, when comparing the AD vs. MCI groups.

#### Interaction between disease status and rFDG

**Fig. 4:**
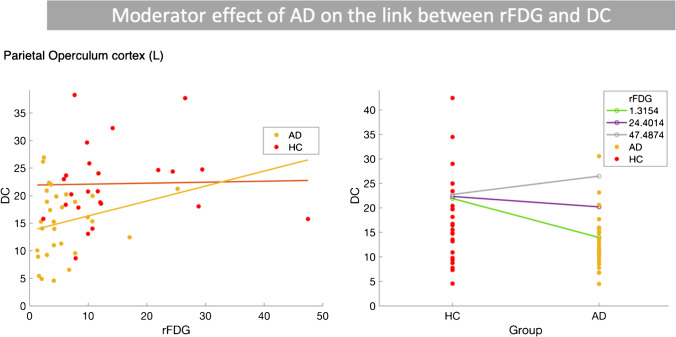
Moderator effect of AD on the link between regional glucose metabolism and inter-regional FC metrics by considering the interaction term between disease status and rFDG in HMMR model. Results showed that only in the left parietal operculum, a significant interaction between disease status and rFDG predicted DC alteration, when comparing AD vs. HC groups (for more information, see Table 4). First column shows that DC decreases as a function of rFDG in HC subjects but increases in AD patients in the left parietal operculum. In second column, we presented that changes in the DC in both HC and AD groups, when the rFDG is fixed at three low, medium and high levels. The lines for HC and AD groups are crossing, indicating an interaction between rFDG and disease status. *AD*, Alzheimer’s disease; *HC*, healthy control; *HMMR*, hierarchical moderated multiple regression; *FC*, functional connectivity; *DC*, degree centrality; *rFDG*, regional glucose metabolism

In the second model, we considered the interaction between disease status and rFDG to test the potential effect of AD on the association between rFDG and CC/DC. Results showed that only in the left parietal operculum a significant interaction between disease status and rFDG predicted DC alteration, when comparing the AD vs. HC groups (Fig. 4; Table 4). The left parietal operculum showed significant hypometabolism and decrease in DC in AD patients compared to HC subjects (SI-Table 4). In Fig. 4, DC showed a negative association with rFDG in HC subjects, but a positive association in AD patients. The significant interaction between rFDG and disease status indicated that the local properties of this region (rFDG and DC) depend on disease status.

**Table 4:** Statistical results related to the second model considering the interaction between disease status and rFDG to predict DC changes, when comparing HC and AD groups

Model parameters and statistics		Parietal operculum cortex (L)
**HMMR model** ^**a,b**^	*R* ^2^	0.93
	Adjusted *R*^2^	0.92
	*F*(5,53)	130.89
	*P*-value_model (FWE-corrected)_	< 0.0001
	$\Delta {R}^{2}$: *R*^2^ change	0.69
	*p*-value_interaction term_	< 0.0001
**Standardized coefficients ** ^**a**^	Disease status	− 0.74
	Age	− 0.11
	Sex	− 0.07
	rFDG	− 0.20
	Interaction (disease status $\times$ rFDG)	0.91

^a^Response variable: DC

^b^Predictors: (constant), rFDG, sex, age, disease status, interaction (disease status $\times$ rFDG)

Abbreviations: *AD*, Alzheimer’s disease; *HC*, healthy control; *HMMR*, hierarchical moderated multiple regression; *DC*, degree centrality; *rFDG*, regional glucose metabolism

## Discussion

We examined the moderator effect of disease (i.e., MCI/AD) on the coupling between regional neural activity and topological measures of inter-regional FC. Our findings demonstrated the following: (i) cortical hypometabolism along with reduced CC/DC in several brain regions including the main hubs of DMN in MCI and AD patients, although in both patients groups, DC was increased in the right parahippocampal gyrus (Fig. 1); (ii) significant two-way interactions between inter-regional FC metrics and “disease status” predicted rFDG alterations in various brain regions in the left hemisphere including parahippocampal gyrus, angular gyrus, caudate, supramarginal gyrus, frontal pole, inferior temporal gyrus, middle frontal, lateral occipital, supramarginal gyrus, precuneus, and the bilateral thalamus, when comparing the HC and AD groups (Figs. 2, 3); (iii) a significant two-way interaction between DC and disease status predicted rFDG alterations in the bilateral thalamus, when comparing the HC and MCI groups (SI-Fig. 2); (iv) a significant two-way interaction between rFDG and disease status predicted DC alterations only in the left parietal operculum, when comparing the HC and AD groups (Fig. 4). These findings suggest that AD disrupts a coupling between rFDG and topological measures of inter-regional FC in particular regions including the main hubs of DMN.

### Hypometabolism and disrupted inter-regional FC topology in MCI/AD

Hypometabolism and FC disruption, mainly in the posterior part of DMN, are well-documented features of AD that are suggested to be strongly linked with grey matter atrophy, amyloid beta deposition, and tau accumulation [2, 3, 17]. Moreover, structural topological changes of several cortical areas within and outside the DMN have been reported in MCI and AD patients [32]. In particular, the authors reported lower nodal clustering and closeness centrality in AD patients in the hippocampus and amygdala, as well as in the parietal, entorhinal, and orbitofrontal areas [32]. In the present study, although both rFDG and CC reductions were congruent along the trajectory of AD (i.e., HC > MCI > AD), DC results were incongruent, namely decreased in several non-DMN areas and increased mainly in the parahippocampal gyrus in both the MCI and AD groups. The parahippocampal gyrus is part of the medial temporal lobe, which has an inverse U pattern of changes in local metabolism along AD trajectory [33]. This pathological hyperactivity in the medial temporal lobe might precipitate the age-associated tau deposition in this region, leading to a loss of white matter integrity and structural dysconnectivity between the temporal lobe and posteromedial cortex [34]. It has been hypothesized that amyloid beta accumulation in the posteromedial cortex disrupts its long-range FC with the medical temporal lobe, which in turn gives rise to disinhibition-like pattern and increased local metabolism in the medical temporal lobe including the hippocampal formation and parahippocampal gyrus. These alterations enhance regional amyloid and tau deposition in the medial temporal lobe, further FC disruption to the remote areas, and finally lead widespread atrophy [7, 8, 35]. Interestingly, normalizing hippocampal hyperactivity, measured by task fMRI, improves task-related memory performance and has a therapeutic potential in MCI patients [36].

### AD decoupled regional neural activity and inter-regional FC topology

We found that AD (as a clinical diagnosis), but not MCI, altered the physiological coupling between CC and rFDG in the left caudate, parahippocampal gyrus, angular gyrus, supramarginal gyrus, frontal pole, and inferior temporal gyrus (Fig. 2(a-f); Table 2). Moreover, both MCI and AD altered the coupling between DC and rFDG in the bilateral thalamus, left middle frontal, left lateral occipital, left supramarginal gyrus, and the left precuneus (Fig. 3(a-f) and SI-Fig. 2; Table 3 and SI-Table 5). Using an alternative model, we observed that interaction between AD and rFDG predicted DC alteration only in the left parietal operculum (Fig. 4; Table 4). These findings collectively indicated that AD interacts with the link between CC/DC and rFDG. The altered association between rFDG and inter-regional FC in AD has been reported previously. For example, Marchitelli and colleagues assessed the link between FDG-PET and different rs-fMRI measures (i.e., fractional amplitude of low-frequency fluctuations (fALFF), regional homogeneity, and group independent component analysis (ICA)) on patients with MCI/AD and also HC subjects using Spearman partial correlations. The correlation between FDG and ICA maps revealed the highest overlap in the posterior part of DMN, while FDG-fALFF correlation pattern showed the lowest overlap, mainly in the frontal and non-DMN regions including the thalamus and caudate nucleus, suggesting rFDG-FC decoupling within and outside DMN areas in MCI/AD patients [15]. Another study demonstrated that local amyloid beta pathology might be responsible for such decoupling between rFDG and inter-regional FC within the DMN in AD [37]. In particular, Scherr and colleagues found that rFDG progressively decoupled from FC in the posterior DMN and degree of this decoupling associated with amyloid beta load. The authors identified “rFDG-FC coupling” as the only significant variable, which predicted cognitive status of the patients with early and late MCI and AD [37]. Similarly, our results support their findings by showing the adverse effect of AD on the normal coupling between rFDG and CC in the DMN. We found widespread and congruent alterations in rFDG and inter-regional FC metrics in the main hubs of DMN including the parahippocampal gyrus, precuneus, angular gyrus, and the inferior temporal gyri, suggesting that AD pathology in the DMN can be (at least partly) accountable for the changes observed in our current study. A longitudinal study demonstrated that severity of amyloid deposition in the regions with high degree of amyloid beta pathology (i.e., in the posterior DMN) can predict progressive hypometabolism in remote but functionally connected areas, with minimal amyloid pathology [38]. It is worthy to mention that amyloid beta and tau pathology interact in their regulation of synaptic function. Synaptic tau and amyloid deposition mutually precipitate signaling pathways that culminate in progressive synaptic dysfunction and loss [39, 40]. Indeed, it has been demonstrated that in individuals with normal amyloid beta level, FC has an inverse correlation with degree of tau deposition [41]. A similar model demonstrated that cascading network failure is mediated by amyloid deposition in the DMN along with global tau deposition [42]. Based on these findings and a recently proposed model by our group [7], it seems that amyloid beta and tau pathology might be important driving forces for alterations in rFDG, inter-regional FC metrics, and rFDG-FC decoupling in AD, which should be directly more elaborated in the future.

In addition, we identified that AD altered the coupling between DC and rFDG in non-DMN areas. Importantly, the important roles of caudate, thalamus, and parieto-occipital cortex have been previously highlighted in AD [43–46]. We assume that tau pathology might be responsible for rFDG and topology alterations beyond the DMN including the thalamus, supramarginal gyrus, and parietal and occipital areas. This is perhaps supported by the fact that tau deposition might be more strongly associated with rFDG decline than amyloid deposition [47]. Moreover, connectivity analysis based on tau imaging showed moderate spatial overlap, not only within the DMN, but also within the visual and language networks [48].

### Strengths and limitations

A major strength of the current study was using hybrid PET/MR scanner, which enabled us to measure both FDG-PET and rs-fMRI at the same time and provided a great opportunity to test the moderator effect of disease on the association between rFDG and inter-regional FC metrics. An important drawback of the current study, however, was the absence of amyloid and tau imaging, which could help to assess the credibility of our hypothesis regarding the role of AD pathology on rFDG and topological measures of inter-regional FC coupling. Thereby, we find it imperative for the next studies to test the pathogenic role of amyloid/tau on metabolism-topology decoupling through simultaneous in vivo amyloid and tau PET imaging. Other limitations were the small samples and age gap between patients and HC subjects. Although we corrected the effect of age by adding it as covariate of no-interest in our regression models, the residual effect of age may still exist or has not-linear effects on functional brain networks [49]. Last but not least, our design was cross sectional, which could impede the drawing of a causal relationship between rFDG and topology changes in AD.

## Conclusion

The present study using simultaneous evaluation of FDG-PET and rs-fMRI provided evidence regarding the role of AD on decoupling between regional neural activity and inter-regional FC alterations. In particular, we demonstrated not only regional metabolism and inter-regional FC were disrupted in patients with MCI and AD, but also there is an adverse effect of AD on coupling between them, within, and outside the DMN. The observed abnormal neuroenergetic coupling of glucose metabolism and inter-regional FC across various brain regions/networks along the trajectory of AD provided novel insights into the pathophysiology of AD. Future longitudinal studies with larger sample size should further test the role of pathological biomarkers of AD (e.g., amyloid and tau proteins) on coupling between regional metabolism and functional dysconnectivity along the trajectory of disease.

## Supplementary Information

Below is the link to the electronic supplementary material.

Supplementary file1 (DOCX 7327 KB)

## Data Availability

The datasets generated during and/or analyzed during the current study are not publicly available due to ethical considerations, but will be available from the corresponding author on reasonable request.

## Code availability

Code can be shared upon reasonable request from the corresponding author.
