# Supplementary material for: Decoupling of regional neural activity and inter-regional functional connectivity in Alzheimer’s disease: a simultaneous PET/MR study
Source: Eur J Nucl Med Mol Imaging. 2022 Feb 24;49(9):3173–85. doi: 10.1007/s00259-022-05692-1 (PMC9250470; doi:10.1007/s00259-022-05692-1)
Supplement: Supplementary file 1 — Supplementary file1 (DOCX 7327 KB) [file 259_2022_5692_MOESM1_ESM.docx]

### Supplementary Information (SI)

**Decoupling of inter-regional functional connectivity and regional neural activity in Alzheimer’s Disease– a simultaneous PET/MRI study**

**Methods:**

**Section1. Imaging data acquisition and preprocessing**

**Data acquisition**

All imaging data were scanned on an integrated Siemens Biograph mMR scanner (Siemens, Erlangen, Germany) capable of simultaneously acquiring PET and MRI data using the vendor-supplied 12-channel phase-array head coil. Magnetization-prepared rapid-acquisition gradient echo (MPRAGE) T1-weighted anatomical images, T2-weighted echo planar imaging MRI data and PET images were acquired using the following scanning parameters. *MP-RAGE*: repetition time/echo time/$\alpha$ = 2.300 ms/2.98 ms/9°; 160 slices (gap 0.5 mm) covering the whole brain; field of view (FOV) 256 mm; matrix size 256 $\times$ 256; voxel size 1.0 $\times$ 1.0 $\times$ 1.0 mm^3^. *Echo planar imaging*: repetition time/echo time/$\alpha$ = 2.000 milliseconds (ms)/30 ms/90°; 35 slices (gap 0.6 mm) aligned to AC/PC covering the whole brain; FOV 192 mm; matrix size 64 $\times$ 64; voxel size 3.0 $\times$ 3.0 $\times$ 3.0 mm^3^. Each measurement consists of 240 acquisitions in interleaved mode with a total scan time of 8 minutes, 08 seconds. *PET*: list-mode acquisition, 30 minutes after injection, 15 minutes acquisition time, 128 slices (gap 0.5 mm) covering the whole brain; FOV 450 mm; matrix size 192 $\times$ 192; voxel size: 3.7 $\times$ 2.3 $\times$ 2.7 mm^3^.

**Preprocessing of imaging data**

Preprocessing of multimodal imaging data started with realigning and coregistering of the multimodal imaging dataset to create subject-specific multimodal datasets. Imaging data were preprocessed using Statistical Parametric Mapping (SPM12) and the Data Processing Assistant for Resting-State fMRI toolbox (DPARSF, V5.1) [1].

To do so, raw DICOM imaging data were converted to 3D nifti volumes and three volumes of each subject’s functional images were then removed for blood oxygen level dependent (BOLD) signal counterbalance, and the remaining volumes were realigned to the same subject’s mean functional image using a least squares approach with a 6 rigid body parameter spatial transformation to estimate head motion of subjects during fMRI scan. Excessive head motion (cumulative motion translation or rotation > 2 mm or 2°) was used as an exclusion criterion. Eight subjects (3 patients with AD, 3 patients with MCI and 2 HC) were excluded under this criterion. Then, mean PET image and the mean functional image and all the functional volumes of the same subject were realigned to the structural T1-weighted image using rigid-body transformation to create subject-specific multimodal datasets.

*MRI images:* The subject-specific T1-weighted images were segmented to tissue-probability maps of grey matter (GM), white matter (WM), and cerebrospinal fluid (CSF) based on affine regularization procedure. The EPI and T1-weighted images were then spatially normalized to a standard template provided by the Montreal Neurological Institute (MNI) template using the Statistical Parametric Mapping (SPM12) unified segmentation on T1-weighted routine. The MNI tissue-probability maps of GM, WM, and CSF were warped onto the single-subject T1-weighted images and stored as masks for later use during the nuisance covariate regression. All the EPI images were resampled to an isotropic voxel size of 3 × 3 × 3 mm^3^. The EPI images were smoothed by the use of Gaussian filter (full-width at half-maximum (FWHM) Gaussian kernel of 4 mm). In order to prepare the rs-fMRI data for functional analysis, they were detrended to remove linear trend [2]. As low frequency (0.01~0.1 Hz) fluctuations of BOLD signal reflects spontaneous neural activity [3], bandpass filtering was also applied on rs-fMRI data to extract physiological signal frequency. Finally, the nuisance variables, such as motion parameters using the Friston-24 model, as well as WM and CSF signals, were regressed out to eliminate their confounding effects [4, 5]. We opted out global signal regression corrections, since this issue is currently challenging in the rs-fMRI field and introduce anti-correlations to data [6].

*FDG-PET images:* The subject-specific PET images were corrected for partial volume effect by the use of PMOD software package (PMOD Technologies Ltd., Adliswil, Switzerland), as suggested previously [7]. This software uses the segmented the individual T1-weighted image in GM, WM, and CSF for partial volume correction (PVC). The PVC-PET images were normalized to the MNI template and resampled to an isotropic voxel size of 3 × 3 × 3 mm^3^ using SPM12. Finally, the PET images were smoothed by the use of Gaussian filter (FWHM Gaussian kernel 12 × 12 × 12 mm^3^).

**Section2: Inter-regional FC topological metrics**

To quantify the organization of the whole brain functional connectivity (FC), we used the topological metrics, which were calculated by graph theory analyses. These included weighted CC [8] and weighted DC [9] metrics, reflecting regional segregation [10] and centrality [11] of FC patterns, respectively. Weighted DC represents the sum of the weights of all edges that are directly linked to a node [9], and is defined as:

${DC}_{i}^{w}=\sum_{j=1}^{N} w_{ij}$ (1)

where $w_{ij}$ is the weight of connection between node i and j, while N is the number of nodes in the graph. The weighted CC for a given node i is equivalent to the fraction of the node neighbors that are also neighbors of each other and is calculated as following:

${CC}_{i}^{w}=\frac{\sum_{j,h\in N} ({w_{ij}w_{jh}w_{ih})}^{1/3}}{{DC}_{i}^{w}({DC}_{i}^{w}-1)}$ (2)

where ${DC}_{i}^{w}$ is the weighted degree of node i, while N is the number of nodes in the graph.

**Section 3: Hierarchical moderated multiple regression (HMMR)**

To understand the moderating effect of disease on the associations between rFDG and the inter-regional FC topological metrics, we used hierarchical moderated multiple regression (HMMR). In this model, the moderation variable is simply the third variable (M) which has an effect on the link between a predictor variable (X) and outcome variable (Y). The term "moderation effect" is equal to the interaction effect in regression analyses. Put differently, a moderator variable potentially changes the strength of an association between two other variables. SI-Figure 1 (A and B) illustrates two most common forms of moderation effects: conceptual diagram and statistical diagram. In particular, hierarchical moderated multiple regression (HMMR) analysis is an analytical approach to investigate the effect of moderator variable M on the association between hypothetical variables X and Y, which can be formulated as the following:

$Y=b_{0}+b_{1}X+b_{2}M+b_{3}\left( X*M \right)+e$ (3)

In this model, $b_{0}$ is an intercept of the regression of Y on X that depends on the specific value of M. There is a different line with different slope and intercept for every individual value of M (SI-Figure 1C). The moderation (interaction) effect is modelled by the (X*M) term, which is the product of X and M. The $b_{3}$ coefficient reflects the interaction between X and M only if the lower order terms $b_{1}X$and $b_{3}M$ are included in the equation**.** To evaluate whether a two-way interaction is significantly present, we first explored whether the increment in the squared multiple correlation (∆R2) given by the interaction is significantly greater than zero. Then, we tested whether the coefficient $b_{3}$ differs significantly from zero.

**SI- Figures:**


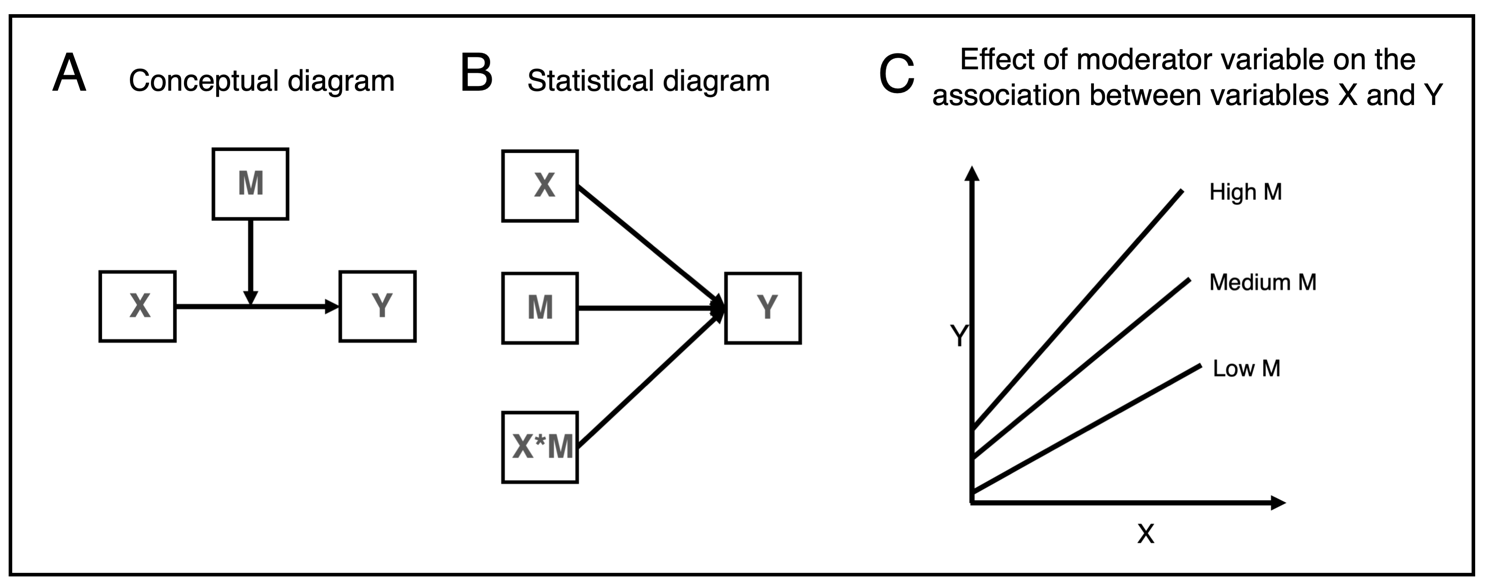


**SI-Figure 1.** Schematic diagrams of moderation are illustrated as A) conceptual diagram and B) statistical diagram. C) The effect of moderator variable on the association between variable X and Y. The slop and intercept of the regression of Y on X that depends on the specific value of M (High, Medium, or Low).


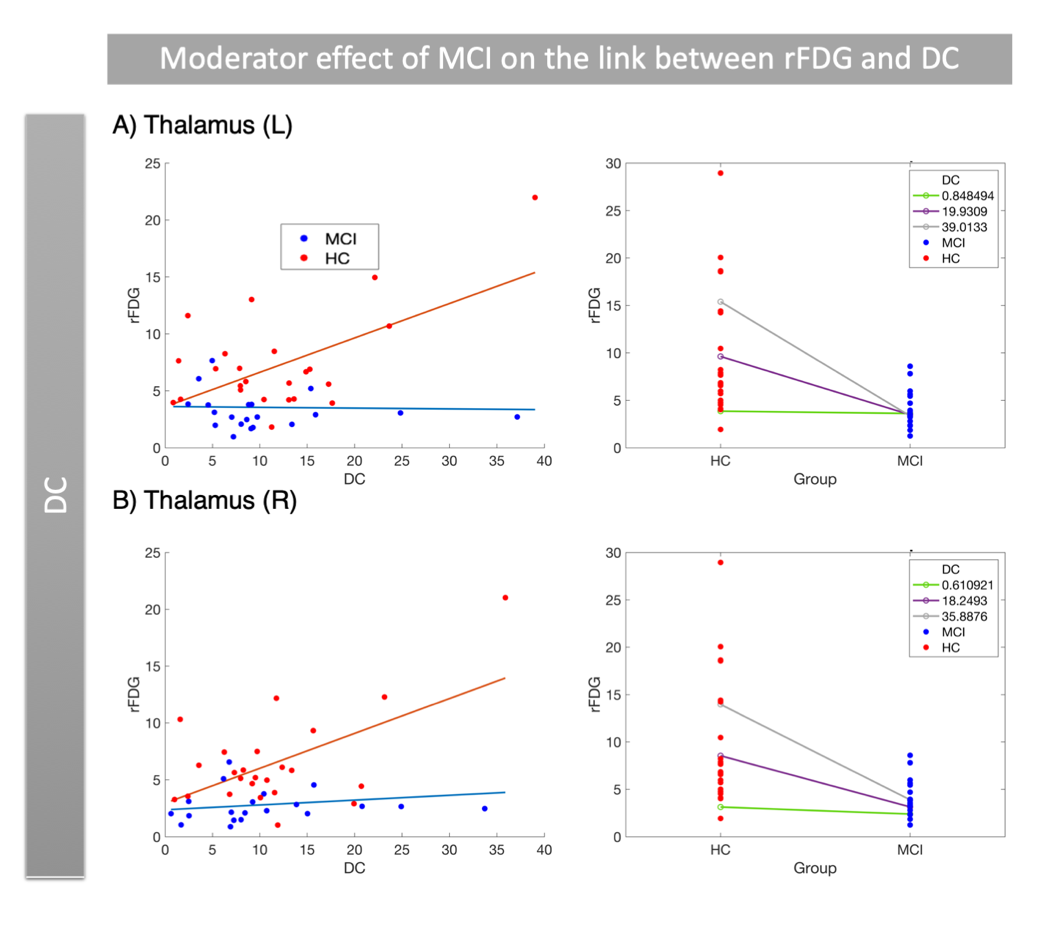


**SI-Figure 2.** Moderator effect of MCI on the link between regional glucose metabolism and FC-metrics by considering the interaction term between disease status and degree centrality in HMMR model. Results showed that in the bilateral thalamus, there was a significant interaction between disease status and DC predicted rFDG changes (for more information, see SI-Table 5). In each section, figures in the first column indicate that rFDG increases as a function of DC in HC subjects but decreases in MCI patients. In the second column of each section, we showed changes in the rFDG in both HC and MCI groups, when the DC is fixed at three low, medium and high levels. The lines for HC and AD are crossing, indicating an interaction between DC and disease status. AD: Alzheimer’s disease; HC: healthy control; HMMR: hierarchical moderated multiple regression; FC: functional connectivity; DC: degree centrality; rFDG: regional glucose metabolism.

**SI-Table 1.** Analysis of covariance on regional glucose uptake for each region in Harvard-Oxford Atlas. Age and sex were considered as the covariates of no-interest. The results were corrected for multiple comparisons using N-region statistical comparison as described previously [12, 13]. The significance threshold for ANCOVA was calculated using 1/number of ROIs, as: p-value <= 1/112=0.009. Post-hoc test: permutation test (100,000 permutations, the significance threshold for post-hoc test was calculated using 1/number of tests, as p-value <= 1/(45$\times$3) <= 0.007).

| **Brain Regions** | **F-test** | **HC** | **MCI** | **AD** | **Post-hoc** | |
| --- | --- | --- | --- | --- | --- | --- |
|  | **p-value** | **Mean**  **(SD)** | **Mean**  **(SD)** | **Mean**  **(SD)** | **Group comparison** | **p-value** |
| Accumbens (L) | 0.0025 | 13.3  (8.1) | 6  (3) | 5.7  (4.6) | HC > AD | 0.0013 |
| Accumbens (R) | 0.0042 | 13.5  (8) | 6.4  (3) | 6  (4.9) | HC > AD | 0.0024 |
| Angular Gyrus (L) | 0.0005 | 13.4  (9.5) | 5.5  (2.8) | 4.6  (4.3) | HC > MCI  HC > AD | 0.0034  0.0001 |
| Angular Gyrus (R) | 0.0048 | 13.2  (9.5) | 5.5  (2.7) | 5.1  (6.3) | HC > MCI  HC > AD | 0.0038  0.002 |
| Brain-Stem (L) | 0.0055 | 2.2  (1) | 1.3  (0.4) | 1.3  (0.6) | HC > AD | 0.0028 |
| Brain-Stem (R) | 0.0080 | 2.3  (0.9) | 1.4  (0.5) | 1.3  (0.6) | HC > AD | 0.0028 |
| Caudate (L) | 0.0006 | 9.3  (6) | 3.6  (1.9) | 3.5  (2.5) | HC > MCI  HC > AD | 0.0027  0.0002 |
| Caudate (R) | 0.002 | 9.2  (5.9) | 3.8  (1.9) | 3.7  (2.8) | HC > MCI  HC > AD | 0.0056  0.0013 |
| Central Opercular Cortex (L) | 0.0082 | 15  (10.4) | 6.2  (3.1) | 6.1  (6) | HC > AD | 0.0042 |
| Cingulate Gyrus, posterior division (L) | 0.0067 | 12.8  (8.5) | 5.7  (2.5) | 5.2  (5.4) | HC > AD | 0.0032 |
| Cingulate Gyrus, posterior division (R) | 0.0063 | 12.5  (8.5) | 5.7  (2.8) | 5.2  (5.3) | HC > AD | 0.0029 |
| Frontal Operculum Cortex (L) | 0.0060 | 15.7  (11.1) | 6.5  (3.5) | 6.1  (5.8) | HC > AD | 0.0027 |
| Frontal Operculum Cortex (R) | 0.0082 | 16.1  (11) | 7  (3.4) | 6.4  (6.2) | HC > AD | 0.0035 |
| Frontal Pole (L) | 0.0063 | 9.4  (6.5) | 4.3  (2) | 3.9  (2.9) | HC > AD | 0.0026 |
| Inferior Temporal Gyrus, posterior division (L) | 0.0006 | 8.3  (5.2) | 3.5  (2) | 2.7  (2.9) | HC > AD | 0.0002 |
| Inferior Temporal Gyrus, posterior division (R) | 0.0017 | 7.4  (4.4) | 3.2  (2.2) | 2.7  (3.1) | HC > MCI  HC > AD | 0.0069  0.0007 |
| Inferior Temporal Gyrus, temporooccipital part (L) | <0.0001 | 11.3  (6.6) | 5  (2.4) | 3.6  (2.5) | HC > MCI  HC > AD | 0.0037  0 |
| Inferior Temporal Gyrus, temporooccipital part (R) | <0.0001 | 10.4  (6.1) | 4.3  (2.2) | 3.5  (2.5) | HC > MCI  HC > AD | 0.0016  <0.0001 |
| Lateral Occipital Cortex, inferior division (L) | 0.0028 | 10.2  (6.3) | 4.6  (2.4) | 4  (4.6) | HC > MCI  HC > AD | 0.0049  0.0013 |
| Lateral Occipital Cortex, inferior division (R) | 0.0086 | 10.5  (6.6) | 4.6  (2.2) | 4.5  (5.9) | HC > MCI  HC > AD | 0.002  0.0058 |
| Lateral Occipital Cortex, superior division (L) | 0.0013 | 11.7  (8.7) | 4.8  (2.6) | 4  (3.9) | HC > MCI  HC > AD | 0.0066  0.0003 |
| Lateral Occipital Cortex, superior division (R) | 0.0052 | 11.5  (8.5) | 4.7  (2.5) | 4.3  (5.2) | HC > MCI  HC > AD | 0.005  0.0019 |
| Middle Frontal Gyrus (L) | 0.0037 | 12  (9) | 5  (2.6) | 4.7  (3.6) | HC > AD | 0.001 |
| Middle Temporal Gyrus, anterior division (L) | 0.0038 | 10.1  (6.6) | 4.8  (2.5) | 4.1  (3.4) | HC > AD | 0.0015 |
| Middle Temporal Gyrus, anterior division (R) | 0.0059 | 8.9  (5.9) | 4.1  (2.3) | 3.8  (3.1) | HC > AD | 0.0027 |
| Middle Temporal Gyrus, posterior division (L) | 0.0001 | 11.2  (7.3) | 5.1  (2.6) | 4.2  (3.9) | HC > AD | 0.0003 |
| Middle Temporal Gyrus, posterior division (R) | 0.005 | 10.9  (7.3) | 4.8  (2.5) | 4.4  (4.5) | HC > AD | 0.0021 |
| Middle Temporal Gyrus, temporooccipital part (L) | 0.0004 | 11.6  (7.4) | 5.2  (2.6) | 4.3  (4.1) | HC > MCI  HC > AD | 0.0032  <0.0001 |
| Middle Temporal Gyrus, temporooccipital part (R) | 0.0025 | 11.3  (7.3) | 5  (2.6) | 4.6  (5.3) | HC > MCI  HC > AD | 0.0021  0.0011 |
| Parahippocampal Gyrus, anterior division (L) | 0.0089 | 7.6  (4.8) | 3.4  (1.9) | 3.1  (3.5) | HC > AD | 0.004 |
| Parietal Operculum Cortex (L) | 0.0077 | 14.3  (10.4) | 5.8  (3.1) | 5.6  (5.1) | HC > AD | 0.0035 |
| Planum Temporale (L) | 0.0052 | 12.4  (8.3) | 5.5  (2.7) | 5  (4.5) | HC > AD | 0.0024 |
| Precuneous Cortex (L) | 0.0076 | 16.7  (11.5) | 7.1  (3.1) | 6.6  (7.3) | HC > AD | 0.004 |
| Subcallosal Cortex (L) | 0.0088 | 11.5  (7.6) | 5.1  (2.8) | 4.7  (4.4) | HC > AD | 0.004 |
| Subcallosal Cortex (R) | 0.0088 | 11  (6.9) | 4.9  (2.7) | 4.6  (4.4) | HC > AD | 0.0045 |
| Superior Parietal Lobule (L) | 0.0009 | 11.2  (8.5) | 4.5  (2.5) | 3.8  (2.8) | HC > AD | 0.0003 |
| Superior Parietal Lobule (R) | 0.0055 | 10.8  (8.4) | 4.3  (2.4) | 3.9  (3.9) | HC > AD | 0.0018 |
| Superior Temporal Gyrus, posterior division (L) | 0.003 | 12.5  (8.6) | 5.5  (2.7) | 4.8  (4.5) | HC > AD | 0.0011 |
| Supramarginal Gyrus, anterior division (L) | 0.0042 | 11.4  (8.2) | 4.7  (2.4) | 4.5  (4.1) | HC > AD | 0.002 |
| Supramarginal Gyrus, posterior division (L) | 0.0016 | 12.8  (9.3) | 5.3  (2.7) | 4.6  (4.5) | HC > MCI  HC > AD | 0.0065  0.0007 |
| Temporal Fusiform Cortex, posterior division (L) | 0.003 | 10.6  (6.7) | 4.8  (2.4) | 4.1  (3.6) | HC > AD | 0.0011 |
| Temporal Fusiform Cortex, posterior division (R) | 0.0059 | 9.3  (5.7) | 4.2  (2.3) | 3.8  (3.9) | HC > AD | 0.0026 |
| Temporal Pole (L) | 0.0061 | 7.6  (4.6) | 3.4  (1.8) | 3.2  (2.8) | HC > AD | 0.0034 |
| Thalamus (L) | 0.0035 | 7.4  (4.4) | 3.4  (1.7) | 3.2  (3) | HC > MCI  HC > AD | 0.0035  0.0018 |
| Thalamus (R) | 0.0046 | 6.5  (4.2) | 2.9  (1.59) | 2.9  (2.7) | HC > MCI  HC > AD | 0.0029  0.0027 |

AD: Alzheimer’s disease; HC: healthy control; MCI: mild cognitive impairment; SD: standard deviation; L: left; R: right.

**SI-Table 2.** Analysis of covariance on clustering coefficient as an inter-regional FC topological metric for each region in Harvard-Oxford Atlas. Age and sex were considered as the covariates of no-interest. The results were corrected for multiple comparisons using N-region statistical comparison as described previously [12, 13]. The significance threshold for ANCOVA was calculated using 1/number of ROIs, as: p-value <= 1/112=0.009. Post-hoc test: permutation test (100,000 permutations, the significance threshold for post-hoc test was calculated using 1/number of tests, as: p-value <= 1/(38$\times$3) <= 0.009).

| **Brain Regions** | **F-test** | **HC** | **MCI** | **AD** | **Post-hoc** | |
| --- | --- | --- | --- | --- | --- | --- |
|  | **p-value** | **Mean**  **(SD)** | **Mean**  **(SD)** | **Mean**  **(SD)** | **Group comparison** | **p-value** |
| Central Opercular Cortex (L) | 0.0001 | 0.36  (0.1) | 0.28  (0.08) | 0.23  (0.08) | HC > AD  MCI > AD | <0.0001  0.0043 |
| Central Opercular Cortex (R) | 0.0001 | 0.36  (0.1) | 0.27  (0.08) | 0.22  (0.08) | HC > AD  MCI > AD | <0.0001  0.009 |
| Cuneal Cortex (R) | 0.0049 | 0.39  (0.13) | 0.37  (0.13) | 0.28  (0.11) | MCI > AD | 0.0007 |
| Frontal Operculum Cortex (R) | 0.0028 | 0.35  (0.11) | 0.29  (0.09) | 0.23  (0.08) | HC > AD  MCI > AD | <0.0001  0.0051 |
| Frontal Pole (R) | 0.0024 | 0.26  (0.08) | 0.25  (0.07) | 0.19  (0.05) | HC > AD  MCI > AD | 0.008  0.0006 |
| Heschls Gyrus (includes H1 and H2) (L) | 0.0023 | 0.36  (0.12) | 0.29  (0.09) | 0.24  (0.1) | HC > AD  MCI > AD | 0.0013  0.0053 |
| Heschls Gyrus (includes H1 and H2) (R) | <0.0001 | 0.38  (0.13) | 0.27  (0.08) | 0.23  (0.08) | HC > AD | <0.0001 |
| Inferior Frontal Gyrus, pars opercularis (R) | 0.0068 | 0.33  (0.09) | 0.27  (0.09) | 0.22  (0.08) | HC > AD | 0.002 |
| Inferior Frontal Gyrus, pars triangularis (L) | 0.0009 | 0.33  (0.12) | 0.29  (0.1) | 0.21  (0.08) | HC > AD  MCI > AD | 0.0022  0.0004 |
| Inferior Temporal Gyrus, temporooccipital part (L) | 0.0018 | 0.33  (0.13) | 0.23  (0.09) | 0.21  (0.09) | HC > AD | 0.0003 |
| Inferior Temporal Gyrus, temporooccipital part (R) | 0.0058 | 0.32  (0.15) | 0.27  (0.08) | 0.21  (0.09) | HC > AD | 0.0018 |
| Insular Cortex (R) | 0.0016 | 0.34  (0.1) | 0.27  (0.08) | 0.21  (0.09) | HC > AD  MCI > AD | 0.0009  0.0038 |
| Supplementary Motor Cortex (L) | 0.0018 | 0.39  (0.12) | 0.32  (0.08) | 0.26  (0.08) | HC > AD  MCI > AD | 0.0014  0.0023 |
| Supplementary Motor Cortex (R) | <0.0001 | 0.4  (0.12) | 0.31  (0.07) | 0.24  (0.08) | HC > AD  MCI > AD | <0.0001  0.0008 |
| Lateral Occipital Cortex, inferior division (R) | 0.0017 | 0.34  (0.13) | 0.3  (0.07) | 0.24  (0.08) | HC > AD  MCI > AD | 0.0017  0.0007 |
| Middle Frontal Gyrus (L) | 0.0005 | 0.33  (0.12) | 0.29  (0.08) | 0.22  (0.07) | HC > AD  MCI > AD | 0.0012  0.0003 |
| Middle Frontal Gyrus (R) | 0.0004 | 0.31  (0.1) | 0.28  (0.09) | 0.21  (0.07) | HC > AD  MCI > AD | 0.0009  0.0003 |
| Middle Temporal Gyrus, anterior division (R) | 0.0014 | 0.31  (0.12) | 0.24  (0.08) | 0.2  (0.06) | HC > AD  MCI > AD | 0.0005  0.0038 |
| Middle Temporal Gyrus, posterior division (L) | 0.0067 | 0.28  (0.11) | 0.27  (0.07) | 0.21  (0.06) | HC > AD  MCI > AD | 0.0052  0.0004 |
| Paracingulate Gyrus (R) | 0.0085 | 0.29  (0.09) | 0.27  (0.08) | 0.22  (0.07) | MCI > AD | 0.003 |
| Parietal Operculum Cortex (L) | 0.0021 | 0.37  (0.13) | 0.27  (0.07) | 0.23  (0.09) | HC > AD | 0.0005 |
| Parietal Operculum Cortex (R) | 0.0041 | 0.37  (0.12) | 0.3  (0.09) | 0.25  (0.08) | HC > AD  MCI > AD | 0.0021  0.003 |
| Planum Polare (L) | 0.0024 | 0.36  (0.15) | 0.27  (0.1) | 0.22  (0.07) | HC > AD  MCI > AD | 0.001  0.0018 |
| Planum Polare (R) | 0.0004 | 0.35  (0.14) | 0.26  (0.07) | 0.21  (0.07) | HC > AD | <0.0001 |
| Planum Temporale (L) | 0.0029 | 0.35  (0.12) | 0.27  (0.07) | 0.23  (0.08) | HC > AD | 0.0008 |
| Planum Temporale (R) | 0.0002 | 0.38  (0.12) | 0.29  (0.08) | 0.24  (0.08) | HC > AD  MCI > AD | <0.0001  0.0046 |
| Postcentral Gyrus (L) | <0.0001 | 0.38  (0.12) | 0.31  (0.06) | 0.24  (0.08) | HC > AD  MCI > AD | <0.0001  0.0004 |
| Postcentral Gyrus (R) | <0.0001 | 0.39  (0.12) | 0.33  (0.07) | 0.25  (0.09) | HC > AD  MCI > AD | <0.0001  0.0003 |
| Precentral Gyrus (L) | 0.0011 | 0.36  (0.11) | 0.29  (0.05) | 0.25  (0.08) | HC > AD | 0.0003 |
| Precentral Gyrus (R) | 0.0016 | 0.38  (0.13) | 0.29  (0.07) | 0.25  (0.08) | HC > AD  MCI > AD | 0.0007  0.0072 |
| Superior Parietal Lobule (L) | 0.0002 | 0.37  (0.1) | 0.31  (0.08) | 0.25  (0.09) | HC > AD  MCI > AD | 0.0001  0.0036 |
| Superior Parietal Lobule (R) | 0.0001 | 0.39  (0.12) | 0.32  (0.07) | 0.25  (0.1) | HC > AD  MCI > AD | 0.0001  0.0016 |
| Superior Temporal Gyrus, posterior division (L) | 0.0036 | 0.32  (0.12) | 0.27  (0.07) | 0.21  (0.06) | HC > AD  MCI > AD | 0.004  0.0006 |
| Superior Temporal Gyrus, posterior division (R) | 0.0025 | 0.33  (0.11) | 0.25  (0.07) | 0.21  (0.06) | HC > AD  MCI > AD | 0.001  0.0048 |
| Supramarginal Gyrus, anterior division (L) | 0.0068 | 0.36  (0.12) | 0.29  (0.08) | 0.24  (0.09) | HC > AD | 0.0021 |
| Supramarginal Gyrus, anterior division (R) | 0.0074 | 0.38  (0.12) | 0.31  (0.11) | 0.24  (0.09) | HC > AD  MCI > AD | 0.0036  0.0074 |
| Supramarginal Gyrus, posterior division (R) | 0.003 | 0.33  (0.11) | 0.26  (0.07) | 0.22  (0.08) | HC > AD | 0.0008 |
| Temporal Occipital Fusiform Cortex (R) | 0.0007 | 0.35  (0.1) | 0.26  (0.08) | 0.24  (0.07) | HC > AD | <0.0001 |

AD: Alzheimer’s disease; HC: healthy control; MCI: mild cognitive impairment; SD: standard deviation; L: left; R: right.

**SI-Table 3.** Analysis of covariance on degree centrality as an inter-regional FC topological metric for each region in Harvard-Oxford Atlas. Age and sex were considered as the covariates of no-interest. The results were corrected for multiple comparisons using N-region statistical comparison as described previously [12, 13]. The significance threshold for ANCOVA was calculated using 1/number of ROIs, as: p-value <= 1/112=0.009. Post-hoc test: permutation test (100,000 permutations, the significance threshold for ANCOVA was calculated using 1/number of test, as: p-value <= 1/(14$\times$3) <= 0.02).

| **Brain Regions** | **F-test** | **HC** | **MCI** | **AD** | **Post-hoc** | |
| --- | --- | --- | --- | --- | --- | --- |
|  | **p-value** | **Mean**  **(SD)** | **Mean**  **(SD)** | **Mean**  **(SD)** | **Group comparison** | **p-value** |
| Amygdala (L) | 0.0032 | 9.9  (5.6) | 6.5  (3.7) | 9.6  (5.5) | HC > MCI  MCI < AD | 0.0002  0.019 |
| Brain-Stem (L) | 0.0064 | 6.3  (6.7) | 5.7  (4.5) | 9.9  (5.8) | MCI < AD | 0.0005 |
| Central Opercular Cortex (R) | 0.0012 | 24.3  (9.9) | 16.2  (7.7) | 14.4  (6.3) | HC > AD | 0.0002 |
| Cingulate Gyrus, posterior division (L) | 0.0052 | 15.3  (7.9) | 17.7  (5.1) | 14  (6) | MCI > HC  MCI > AD | 0.0009  0.013 |
| Cingulate Gyrus, posterior division (R) | 0.0004 | 15.8  (9.2) | 19.8  (6.6) | 14.1  (7) | MCI > HC  MCI > AD | 0.0002  0.0015 |
| Insular Cortex (R) | 0.0021 | 20.5  (8.5) | 13.4  (4.6) | 11.8  (5.9) | HC > MCI  HC > AD | 0.0183  0.0009 |
| Lateral Occipital Cortex, inferior division (L) | 0.007 | 21.9  (10.3) | 13.9  (5.9) | 13.9  (6.7) | HC > AD | 0.002 |
| Lateral Occipital Cortex, inferior division (R) | 0.007 | 19.2  (8.6) | 13.3  (5.3) | 13.4  (6.8) | HC > MCI  HC > AD | 0.019  0.002 |
| Middle Frontal Gyrus (R) | 0.0082 | 14.1  (6.5) | 12.4  (6) | 9.6  (5.8) | HC > AD  MCI > AD | 0.002  0.012 |
| Parahippocampal Gyrus, anterior division (R) | 0.0017 | 5.7  (4.8) | 10.3  (6.1) | 10.4  (6) | MCI > HC  AD > HC | 0.0007  0.0009 |
| Parietal Operculum Cortex (L) | 0.0066 | 22  (7.2) | 17.7  (5.2) | 15.2  (6.2) | HC > AD | 0.001 |
| Parietal Operculum Cortex (R) | 0.0067 | 22  (7.7) | 14.6  (6.1) | 13.7  (6.5) | HC > MCI  HC > AD | 0.015  0.001 |
| Planum Polare (R) | 0.002 | 18.8  (7.3) | 12.6  (7.5) | 11.6  (5.2) | HC > MCI  HC > AD | 0.013  0.0003 |
| Superior Parietal Lobule (R) | 0.0012 | 18.3  (7.1) | 14.1  (4.4) | 10.9  (6.6) | HC > AD  MCI > AD | 0.0004  0.017 |

AD: Alzheimer’s disease; HC: healthy control; MCI: mild cognitive impairment; SD: standard deviation; L: left; R: right.

**SI-Table 4.** Overlapping brain regions determined by the direction of change in regional glucose metabolism and inter-regional functional connectivity topology metrics (CC: clustering coefficient, DC: degree centrality)

|  | **Brain regions** | **Regional glucose metabolism**  **(rFDG)** | **Inter-regional functional connectivity topology metrics** | |
| --- | --- | --- | --- | --- |
|  |  |  | **CC** | **DC** |
| MCI vs. HC | Lateral Occipital Cortex, inferior division (R) | HC > MCI | - | HC > MCI |
| AD vs. HC | Central Opercular Cortex (L) | HC > AD | HC > AD | - |
|  | Frontal Operculum Cortex (R) | HC > AD | HC > AD | - |
|  | Inferior Temporal Gyrus, temporooccipital part (L) | HC > AD | HC > AD | - |
|  | Inferior Temporal Gyrus, temporooccipital part (R) | HC > AD | HC > AD | - |
|  | Lateral Occipital Cortex, inferior division (L) | HC > AD | - | HC > AD |
|  | Lateral Occipital Cortex, inferior division (R) | HC > AD | HC > AD | HC > AD |
|  | Middle Frontal Gyrus (L) | HC > AD | HC > AD | - |
|  | Middle Temporal Gyrus, anterior division (R) | HC > AD | HC > AD | - |
|  | Middle Temporal Gyrus, posterior division (L) | HC > AD | HC > AD | - |
|  | Parietal Operculum Cortex (L) | HC > AD | HC > AD | HC > AD |
|  | Planum Temporale (L) | HC > AD | HC > AD | - |
|  | Superior Parietal Lobule (L) | HC > AD | HC > AD | - |
|  | Superior Parietal Lobule (R) | HC > AD | HC > AD | HC > AD |
|  | Superior Temporal Gyrus, posterior division (L) | HC > AD | HC > AD | - |
|  | Supramarginal Gyrus, anterior division (L) | HC > AD | HC > AD | - |
| AD vs. MCI | N.S | | | |

AD: Alzheimer disease; HC: healthy control; MCI: mild cognitive impairment; L: left; R: right; N.S: Not significant.

**SI-Table 5.** Statistical results related to the first model considering the interaction between disease status and DC to predict rFDG changes, when comparing HC and MCI groups.

| Model parameters and statistics | | Thalamus (L) | Thalamus (R) |
| --- | --- | --- | --- |
| HMMR model^a,b^ | R^2^ | 0.53 | 0.50 |
|  | Adjusted R^2^ | 0.46 | 0.43 |
|  | F(5,39) | 7.59 | 6.76 |
|  | P-value_model (FEW-corrected)_ | < 0.0001 | < 0.0001 |
|  | $\Delta R^{2}$: R^2^ Change | 0.11 | 0.07 |
|  | p-value_interaction term_ | 0.01 | 0.04 |
| Standardized Coefficients ^a^ | Clinical_group | 0.03 | -0.05 |
|  | Age | 0.009 | 0.07 |
|  | Sex | -0.22 | -0.20 |
|  | DC | 1.29 | 1.23 |
|  | Interaction  (Clinical_group x DC) | -1.06 | -0.96 |

1. Response variable: rFDG
2. Predictors: DC, Sex, Age, Clinical_group, intraction (Clinical_group x DC)

**References:**

1. Yan CG, Wang XD, Zuo XN, Zang YF. DPABI: Data Processing & Analysis for (Resting-State) Brain Imaging. Neuroinformatics. 2016;14:339-51. doi:10.1007/s12021-016-9299-4.

2. Lowe MJ, Russell DP. Treatment of baseline drifts in fMRI time series analysis. J Comput Assist Tomogr. 1999;23:463-73.

3. Lu H, Zuo Y, Gu H, Waltz JA, Zhan W, Scholl CA, et al. Synchronized delta oscillations correlate with the resting-state functional MRI signal. Proceedings of the National Academy of Sciences of the United States of America. 2007;104:18265-9. doi:10.1073/pnas.0705791104.

4. Kelly AM, Uddin LQ, Biswal BB, Castellanos FX, Milham MP. Competition between functional brain networks mediates behavioral variability. NeuroImage. 2008;39:527-37. doi:10.1016/j.neuroimage.2007.08.008.

5. Satterthwaite TD, Elliott MA, Gerraty RT, Ruparel K, Loughead J, Calkins ME, et al. An improved framework for confound regression and filtering for control of motion artifact in the preprocessing of resting-state functional connectivity data. Neuroimage. 2013;64:240-56. doi:10.1016/j.neuroimage.2012.08.052.

6. Murphy K, Fox MD. Towards a consensus regarding global signal regression for resting state functional connectivity MRI. NeuroImage. 2017;154:169-73. doi:10.1016/j.neuroimage.2016.11.052.

7. Mevel K, Desgranges B, Baron JC, Landeau B, De la Sayette V, Viader F, et al. Detecting hippocampal hypometabolism in Mild Cognitive Impairment using automatic voxel-based approaches. Neuroimage. 2007;37:18-25. doi:10.1016/j.neuroimage.2007.04.048.

8. Saramäki J, Kivelä M, Onnela JP, Kaski K, Kertész J. Generalizations of the clustering coefficient to weighted complex networks.

. Physical review E, Statistical, nonlinear, and soft matter physics. 2007;E 75:027105.

9. Rubinov M, Sporns O. Weight-conserving characterization of complex functional brain networks. Neuroimage. 2011;56:2068-79. doi:10.1016/j.neuroimage.2011.03.069.

10. van den Heuvel MP, Hulshoff Pol HE. Exploring the brain network: a review on resting-state fMRI functional connectivity. Eur Neuropsychopharmacol. 2010;20:519-34. doi:10.1016/j.euroneuro.2010.03.008.

11. Sporns O. Network attributes for segregation and integration in the human brain. Curr Opin Neurobiol. 2013;23:162-71. doi:10.1016/j.conb.2012.11.015.

12. Lynall ME, Bassett DS, Kerwin R, McKenna PJ, Kitzbichler M, Muller U, et al. Functional connectivity and brain networks in schizophrenia. The Journal of neuroscience : the official journal of the Society for Neuroscience. 2010;30:9477-87. doi:10.1523/JNEUROSCI.0333-10.2010.

13. Meng C, Brandl F, Tahmasian M, Shao J, Manoliu A, Scherr M, et al. Aberrant topology of striatum's connectivity is associated with the number of episodes in depression. Brain : a journal of neurology. 2014;137:598-609. doi:10.1093/brain/awt290.
